# The impact of sleep duration, depressive symptoms, and cognitive function on daily activity among Chinese older adults: a serial multiple mediation model

**DOI:** 10.7189/jogh.15.04267

**Published:** 2025-09-12

**Authors:** Jiayi Sun, Hao Li, Jun Deng, Jianing Liu, Nachuan Hu, Hui Liu, Yangdong Fan, Lei Shi

**Affiliations:** 1 School of Health Management, Guangzhou Medical University, Guangzhou, Guangdong, China; 2 Department of Basic Public Health, Guangzhou Center for Disease Control and Prevention (Guangzhou Health Supervision Institute), Guangzhou, China; 3 Institute of Public Health, Guangzhou Medical University & Guangzhou Center for Disease Control and Prevention (Guangzhou Health Supervision Institute), Guangzhou, China; 4 School of Health Management, Guangzhou Medical University, Guangzhou, China; 5 Key Laboratory of Philosophy and Social Sciences of Colleges and Universities in Guangdong Province for Collaborative Innovation of Health Management Policy and Precision Health Service, Southern Medical University, Guangzhou, China; 6 Philosophy and Social Sciences Key Laboratory of Guangdong Higher Education Institutes for Health Governance Based on Big Data Utilization, Guangzhou Medical University, Guangzhou, China; 7 Local Government Development Research Institute of Shantou University, Shantou, China

## Abstract

**Background:**

Few studies have explained the mechanisms underlying the relationship between sleep duration and the activities of daily living (ADL). We aim to explore the multiple mediating roles of depressive symptoms and cognitive function in this relationship among Chinese older adults.

**Methods:**

A total of 5858 older adults participated in the China Health and Retirement Longitudinal Study, completing the Center for Epidemiologic Studies Depression Scale, the ADL Scale, and the Telephone Interview for Cognitive Status Scale. We performed serial multiple mediation analysis using the Hayes’ PROCESS macro.

**Results:**

Sleep duration influenced ADL both directly and indirectly through three significant pathways (*P* < 0.001). For individuals sleeping 6–8 hours, depressive symptoms accounted for 56.50% of the total effect. In contrast, for those sleeping ≥8 hours, depressive symptoms accounted for 65.50%. Cognitive function contributed to 1.79% of the total effect in the 6–8-hour group, whereas in the ≥8-hour group, cognitive function had a negative mediating effect of −4.60%. Combined mediation by depressive symptoms and cognitive function accounted for 2.42% of the total effect in the 6–8-hour group and 2.76% in the ≥8-hour group. The total mediating effect was 60.70% for the 6–8-hour group and 63.70% for the ≥8-hour group.

**Conclusions:**

The action mechanisms between different levels of sleep duration and ADL differed, but all showed significant effects from sleep duration, depressive symptoms, and cognitive function regarding ADL among older adults. Therefore, promoting sleep education and addressing depressive symptoms and cognitive decline in older adults are essential for the early detection and prevention of ADL impairment.

As the global population ages, the resulting ‘silver wave’ presents both opportunities and challenges for economic and social development [1]. In this context, older adults' health has emerged as a crucial factor for sustainable societal development [2]. According to estimates, the number of people aged 65 years and older with impairment in daily living activities will rise to 52.05 million by 2050, which is approximately 2.8 times that of 2020 (18.67 million people). The population of older adults with cognitive impairment will also reach 28.98 million by then [3]. One study demonstrated the importance of sleep to health, especially in older adults with impaired activities of daily living (ADL), concluding that good sleep is essential for maintaining physical and mental health as well as cognitive function in older adults [4]. Older adults may encounter various sleep disorders, such as insomnia and poor sleep quality, as they age. These issues not only affect their mental health and cognitive function but also reduce their ability to perform daily living tasks, which can seriously impact their overall well-being [5]. This situation requires focus on the sleep health of older adults, clarifying the mechanism linking sleep and ADL, and adopting targeted prevention and intervention measures to improve their quality of life.

Recent study results using data from the China Longitudinal Study of Health and Retirement suggest that ADL is closely related to several key factors among the participants, including the personal characteristics, behavioural lifestyle, and health status factors of the study participants [6-8]. These factors specifically include age, sex, education level, chronic diseases, living environment, socioeconomic status, lifestyle, and psychological status [9]. ADL may decrease with age, and factors such as education level, chronic diseases, lifestyle (*e.g.* smoking and exercise), and psychological status can significantly impact ADL [10,11].

Sleep deprivation affects the brain's mood regulation centres, making people more prone to anxiety, irritability, and depression. Chronic sleep deprivation can also lead to mood disorders and other mental health problems [12,13]. Previous studies have shown that sleep duration is closely associated with psychiatric disorders and dementia [14,15]. Other studies have also explored the possible genetic mechanisms and structural brain changes that underlie the nonlinear relationship between sleep duration, cognitive function, and mental health [16,17].

Depression is a common mental health disorder. It affects all aspects of life, particularly cognitive functioning [18,19]. Although depression’s impact varies depending on its severity, duration, treatment, and individual differences, in depression, most studies have demonstrated that its cumulative effects negatively affect cognitive functioning [20,21]. Its duration is associated with cognitive impairment, which may persist even after the remission of depressive symptoms [22].

Cognitive function is one of the most obvious health indicators that changes during the ageing process. According to the life cycle theory, both physical and cognitive function decline with age in older adults [23]. A previous study has shown that cognitive impairment is a strong predictor of ADL and that cognitive decline often precedes the loss of ADL independence [24]. Conversely, improved cognitive functioning may lead to behavioural changes that promote ADL participation in older adults [25]. This suggests that enhancing cognitive functioning and improving older adults’ quality of life may delay the loss of ADL independence.

Overall, previous studies have provided a scientific basis for the pairwise relationships between ADL’s relevant influencing factors [26,27]. Although the association between sleep duration and ADL in older adults has been well documented, room exists for further investigation. First, most studies have focussed on direct effects, ignoring the mediating role of depressive symptoms and cognitive functioning despite evidence of dynamic interactions between these factors. Second, existing mediation analyses have focussed on Western populations; however, genetic, cultural, and lifestyle differences may alter these pathways in the Asian context [28,29]. Third, the nonlinear effects of different sleep duration groups (*e.g.* ≤6, 6–8, *vs.* ≥8 hours) on ADL have not been explored in continuous mediation models. We address the gap in existing research on the interplay between sleep duration, psychological state, and cognitive function in older Chinese adults, as well as their combined impact on daily living ability. We also advance the detailed study of mediation models in this context. Specifically, we explore the mechanisms underlying the relationship between sleep duration and ADL decline in older adults. We introduce depressive symptoms and cognitive function as mediating variables and control for other influencing factors. Finally, we construct a serial multiple mediation model examining the relationship between sleep and daily living ability. We hypothesised that depressive symptoms mediate the relationship between sleep duration and ADL. Second, cognitive function was hypothesised to mediate the relationship between sleep duration and ADL. Third, we hypothesised that depressive symptoms and cognitive function mediate the relationship between sleep duration and ADL (**Figure 1**).

**Figure 1:**
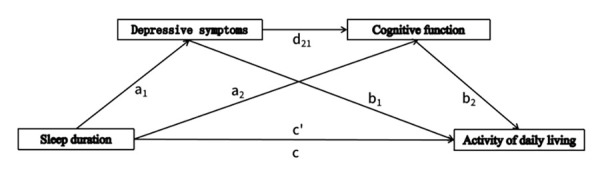
Diagram of the research hypothesis model.

## METHODS

### Participants and ethics statement

We obtained the data from the fifth round (2020) of the China Health and Retirement Longitudinal Study (CHARLS). The CHARLS is a nationally representative cohort study covering 150 county-level and 450 village-level units in China. It comprehensively collects information on multiple aspects, including basic information, health status, functional ability, personal income, household income, and expenditure. The total sample size for this round was 19 395 cases. We were authorised to download the data. The criteria for enrollment included older adults aged ≥60 years and complete responses. Ultimately, 5858 older adults were included in this study (Figure S1 in the **Online Supplementary Document**).

### Measurements

#### Sleep duration

We choose sleep duration as the primary measure of sleep given its quantifiability, data availability, feasibility for statistical analysis, direct relevance to practical applications and interventions, and its use as the basis of previous research [30-33]. In the CHARLS programme, nap duration was assessed by asking, ‘How long was a typical nap in the past 1 month?’. Nighttime sleep duration was assessed by asking, ‘In the past 1 month, about how many hours did you actually fall asleep each night?’. Previous studies have shown that napping and nighttime sleep duration are widely used and have good reliability [34,35]. As sleep in older adults is usually divided into multiple stages, naps can compensate for the lack of nighttime sleep [36]. Among Chinese older adults, daytime naps are the norm, and 64.5% of CHARLS participants take regular naps; therefore, measuring nighttime sleep alone may be misleading [37]. We thus calculated the total sleep duration by summing the durations of naps and nighttime sleep. Guidelines provided by the National Sleep Foundation recommend an optimal sleep time of 7–9 hours for adults and 6–8 hours for seniors. Moreover, considering that the effect of sleep duration on cognitive function differs between 6–8 and 8 hours [36-38], we categorised sleep duration into three groups: ≤6, 6–8, and ≥8 hours.

#### Depressive symptoms

We used the Center of Epidemiological Studies Depression Scale, ten-item version, to assess depressive mood in older adults [39,40]. The scale consists of ten items, each with four response options: 0 = rarely or not at all; 1 = some or rarely; 2 = occasionally or moderately; and 3 = mostly or completely. The total score ranges from 0–30. Questions five and eight are reverse-scored. Scores of ≥10 indicate the presence of depressive symptoms. The Cronbach's alpha coefficient for the scale was 0.846.

#### Cognitive function

We assessed cognitive function using the Telephone Interview for Cognitive Status [41]. The scale measures an individual's cognitive function across two dimensions: memory and psychosomatic states. Memory was measured using immediate (0–10 points) and delayed word recall (0–10 points). The respondents were asked to recall and retell a list of words. They received one point for each word correctly recalled immediately and after a short delay. The mental status was measured in three areas: orientation, visual construction, and math performance. Orientation was evaluated by asking the respondents to name their current date, day of the week, and season, with a maximum score of five points (one point for each correct answer). Visual construction was assessed by having respondents redraw a previously displayed picture, with a score of 0–1 point (one point for successful drawing). Mathematical performance was measured by asking respondents to subtract seven from 100 five times consecutively, with a maximum score of five points (one point for each correct calculation). Cognitive function scores were obtained by summing the scores of the above questions, with the total score ranging from 0–31. Higher scores indicated better cognitive function and cognitive soundness [42]. The Cronbach's alpha coefficient for the scale was 0.696.

#### Activity of daily living

We used two assessment dimensions to comprehensively evaluate ADL in older adults: basic ADL and instrumental ADL [43]. Each dimension comprised six items. Basic ADL include getting dressed, eating, using the toilet, urinating and defecating, bathing, and getting up. Instrumental ADL include making phone calls, cooking, taking medication, shopping, managing money, and doing housework. Each item had four options: 4 = no difficulty; 3 = difficulty but can still be accomplished; 2 = difficulty, need help; and 1 = cannot be accomplished. The combined result yields a total ADL score ranging from 12–48, with lower scores indicating poorer activity. The total Cronbach's alpha coefficient for the scale was 0.917.

#### Control variables

Control variables included demographic background (*i.e.* age, sex (male/female), marital status (married/non-married)), education level (illiterate/elementary/junior high/middle/high school and above); behavioural lifestyles (*i.e.* smoking (yes/no), exercise (top score is three), and socialisation (score range: 0–7)), and health status factors (*i.e.* self-assessed health status (excellent/good/fair/poor/very poor), chronic diseases (yes/no)). Chronic diseases in the CHARLS survey refer to conditions that a doctor informs respondents about, and the data set uses a ‘yes’ or ‘no’ criterion to determine the status of these diseases.

### Data analysis

We used SPSS, version 26.0 (SPSS Inc., Chicago, Illinois, USA) and PROCESS, version 4.1 [44] to analyse the data. We used Cronbach’s alpha to determine the scales’ internal consistency. After conducting normality tests for ADL, we assessed cognitive ability, depressive symptoms, and sleep duration. We used Pearson correlation to examine the associations between the different continuous variables. We conducted a serial multiple mediation analysis to explore the mediation patterns among sleep duration, depressive symptoms, cognitive functioning, and ADL. We entered sleep duration as an independent variable and ADL as a dependent variable. We identified depressive symptoms and cognitive function as the proposed mediators. The bootstrap sample size for the percentile bootstrap confidence intervals was 5000, using a 95% confidence interval (CI). If the 95% CI for the mediating effect did not contain zero, the effect would be significant at the 0.05 level. We used bootstrapping to test the mediating effects of depressive symptoms and cognitive functioning. To examine the multiple mediating effects of sleep duration (a multicategory independent variable), we employed a serial multiple mediation analysis combined with indicator coding [45]. Specifically, we categorised sleep duration into three levels: ≤6 (reference), 6–8, and ≥8 hours. We then tested the mediating effects of sleep duration separately for the 6–8 and ≥8-hour groups, using ≤6 hours as the reference category. All statistical tests were two-sided, with the statistical significance set at *P* < 0.05.

### Common method biases

Before conducting multiple chain mediation analyses, we tested the severity of the common method bias. Harman's single-factor test is a common method bias test for sleep duration, depression, cognition, and ADL, and has been widely used in common method bias testing [46,47]. After performing principal component analysis, we extracted nine eigenvalues >1. The first factor accounted for 14.767% of the variance among all items of the research variables, reflecting both common method bias and the inherent relationships between the variables. Because this percentage was far below the critical value of 40% [47], we concluded that common method bias was not a serious concern in this study.

## RESULTS

### Preliminary analyses

The mean age of the participants was 67.6 years (standard deviation = 5.67), and the sample was predominantly male (58.5%) (**Table 1**). Most participants were married (82.6%) and had a low level of education (64.2%). The majority self-assessed their health as average (53.3%). Regarding behavioural lifestyle, most participants did not smoke (61.3%), the mean score for socialisation was 1.29 out of 7, and the mean score for exercise was 1.68 out of 3. The mean total sleep duration was 6.82 hours. The mean depression symptoms score was 8.22 out of 30, with 30.2% exhibiting depressive symptoms. The mean cognitive functioning score was 17.43 out of 31. The mean score for the ADL was 46.72 out of 48.

**Table 1:** Demographic characteristics of participants (n = 5858)

	n (%)
Age*	67.62 (5.67)
**Gender**	
Male	3426 (58.5)
Female	2432 (41.5)
**Marital status**	
In marriage	4840 (82.6)
Out of marriage	1018 (17.4)
**Educational level**	
Illiteracy	744 (13.7)
Primary school	2736 (50.5)
Junior middle school	1204 (22.2)
High school and above	1174 (13.6)
**Self-assessment of health status**	
Excellent	596 (10.2)
Good	665 (11.4)
General	3120 (53.3)
Poor	1126 (19.2)
Very poor	351 (5.9)
**Smoking**	
Yes	2265 (38.7)
No	3593 (61.3)
Socialisation*	1.29 (0.67)
Exercise*	1.68 (0.88)
Total hours of sleep*	6.82 (2.06)
Nap duration*	0.77 (0.76)
Nighttime sleep duration*	6.05 (1.80)
Depression score*	8.22 (6.22)
**Depressive symptoms (CES-D-10 score ≥11)**	
Yes	1769 (30.2)
No	4089 (69.8)
Cognitive functioning score*	17.43 (4.93)
ADL score*	46.72 (3.03)

ADL – activities of daily living, CES-D-10 **–** Center for Epidemiologic Studies Depression Scale

*Value presented as mean (standard deviation).

In the correlation matrices for the different variables, sleep duration (*r* = 0.153; *P* < 0.01) and cognitive ability (*r* = 0.157; *P* < 0.01) were significantly positively correlated with ADL (Table S1 in the **Online Supplementary Document**). Depressive symptoms were negatively correlated with ADL (*r* = −0.338; *P* < 0.01). Lastly, cognitive ability (*r* = −0.225; *P* < 0.01) and sleep duration (*r* = −0.273; *P* < 0.01) were negatively correlated with depressive symptoms.

### Serial multiple mediation analysis

Sleep duration X_2_ (a_11_ = −2.997; *P* < 0.001) and X_3_ (a_12_ = −2.893; *P* < 0.001) were negatively correlated with depressive symptoms (**Table 2**). Depressive symptoms were negatively correlated with cognitive function (d_21_ = −0.174; *P* < 0.001). However, sleep duration X_2_ and X_3_ showed opposite effects on cognitive functioning: sleep duration X_2_ was positively correlated with cognitive functioning (a_21_ = 0.395; *P* < 0.001), and sleep duration X_3_ was negatively correlated with cognitive functioning (a_22_ = −0.833; *P* < 0.001). Additionally, depressive symptoms were negatively correlated with ADL (b_1_ = −0.148, *P* < 0.001), and cognitive function was positively correlated with ADL (b_2_ = 0.036; *P* < 0.001). Sleep duration X_2_ (c_1_’ = 0.309; *P* < 0.001) and X_3_ (c_2_’ = 0.237; *P* < 0.01) were positively correlated with ADL.

**Table 2:** Regression coefficients in the serial multiple mediation analysis

Dependent variable	Independent variable	R	R^2^	F	B	95% CI	*t*	*P*-value
Depressive symptoms	X_2_	0.293	0.086	91.339	−2.997	−3.343, −2.651	−16.982	<0.001
	X_3_				−2.893	−3.307, −2.480	−13.712	<0.001
Cognitive function	X_2_	0.290	0.084	76.547	0.395	0.114, 0.676	2.756	<0.001
	X_3_				−0.833	−1.166, −0.499	−4.897	<0.001
	Depressive symptom				−0.174	−0.194, −0.153	−16.747	<0.001
ADL	X_2_	0.393	0.154	133.255	0.309	0.142, 0.475	3.641	<0.001
	X_3_				0.237	0.039, 0.434	2.353	<0.01
	Depressive symptom				−0.148	−0.160, −0.135	−23.549	<0.001
	Cognitive function				0.036	0.020, 0.051	4.610	<0.001

ADL – activities of daily living, CI – confidence interval, X_1_ – sleep duration <6 hours, X_2_ – sleep duration 6–8 hours, X_3_ – sleep duration >8 hours

To test the significance of the mediating effects of depressive symptoms and cognitive function, we conducted a bootstrap estimation procedure with 5000 bootstrap samples (**Table 3**). According to this test, the mediating effect was significant if the 95% CI of the path coefficients did not include zero [48].

**Table 3:** Bootstrap tests for relative indirect effects, total effects, and direct effects

		Bootstrapped distribution			
	**Duration of sleep (X_1_ = 1 as a reference)**	***β***	**95% CI**	***P*-value**	**Ratios of relative effects, %**
**Relative indirect effects**	X_2_ = 2	0.476		<0.05	60.70
	X_3_ = 3	0.415		<0.05	63.70
Ind1	X_2_ = 2	0.443	0.371, 0.52	<0.05	56.50
	X_3_ = 3	0.427	0.352, 0.507	<0.05	65.50
Ind2	X_2_ = 2	0.014	0.004, 0.027	<0.05	1.79
	X_3_ = 3	−0.03	−0.051, −0.013	<0.05	−4.60
Ind3	X_2_ = 2	0.019	0.009, 0.029	<0.05	2.42
	X_3_ = 3	0.018	0.009, 0.028	<0.05	2.76
**Relative direct effects**	X_2_ = 2	0.309	0.142, 0.475	<0.05	39.40
	X_3_ = 3	0.237	0.039, 0.434	<0.05	36.30
**Relative total effects**	X_2_ = 2	0.785	0.613, 0.955	<0.05	
	X_3_ = 3	0.652	0.448, 0.856	<0.05	

CI – bias-corrected confidence interval, Ind1 – the route mediated by depressive symptoms, Ind2 – the route mediated by cognitive function, Ind3 – the route mediated by both depressive symptoms and cognitive function, X_1_ – sleep duration <6 h, X_2_ – sleep duration 6–8 h, X_3_ – sleep duration >8 h

When we included depressive symptoms (c_1_’ = 0.309; *P* < 0.001) and cognitive function (c_2_’ = 0.237; *P* < 0.01) in the model, the direct effect of sleep duration on ADL remained significant. Sleep duration indirectly affects ADL through three significant mediating pathways. The effects of different sleep durations on ADL showed distinct roles in the mediating pathways involving cognitive function. Specifically, sleep duration was categorised into three groups: ≤6, 6–8, and ≥8 hours. With the reference category being X_1_ (≤6 hours), serial multiple mediation analyses were performed for two sleep duration categories: X_2_ (6–8 hours) and X_3_ (≥8 hours).

### Sleep duration of 6–8 hours

Depressive symptoms (*β*_1_ = 0.443; 95% CI = 0.371, 0.520) accounted for 56.50% of the total effect (**Table 3**). These findings suggest that depressive symptoms play a significant role in daily functioning. Cognitive function (*β*_1_ = 0.014; 95% CI = 0.004, 0.027) accounted for 1.79% of the total effect. Both depressive symptoms and cognitive function (*β*_1_ = 0.019; 95% CI = 0.009, 0.028) accounted for 2.42% of the total effect. The total mediating effect was 60.70%.

### Sleep duration ≥8 hours

Depressive symptoms (*β*_2_ = 0.427; 95% CI = 0.352, 0.507) accounted for 65.50% of the total effect. This not only suggests that depressive symptoms play a major role in influencing ADL but also implies that people who sleep too much are more negatively affected by depressive symptoms than those who sleep a normal amount. Cognitive function (*β*_2_ = −0.030; 95% CI = −0.051, −0.013) accounted for −4.60% of the total effect. Both depressive symptoms and cognitive function (*β*_2_ = 0.018; 95% CI = 0.009, 0.028) accounted for 2.76% of the total effect. The total mediating effect was 63.70%.

**Figure 2:**
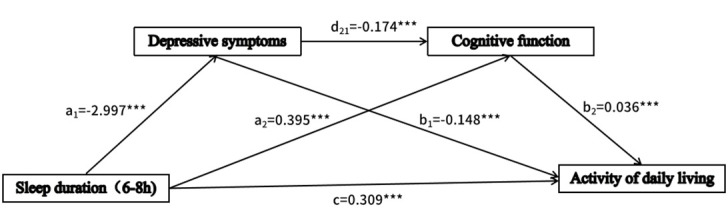
Chain mediation effect model diagram (sleep duration = 6–8 hours). ***P < 0.001.

**Figure 3:**
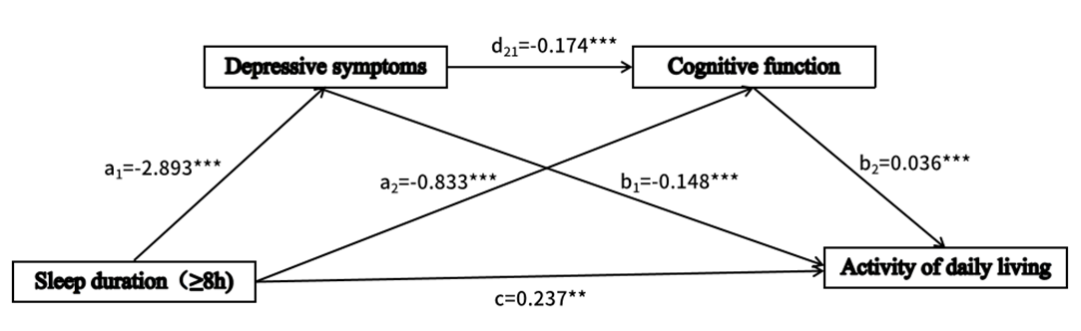
Chain mediation effect model diagram (sleep duration ≥8 hours). ***P < 0.001.

In general, all relationships in the model were statistically significant, with both positive and negative effects. The sleep duration’s direct effect on ADL remained significant after accounting for the mediators of depressive symptoms and cognitive function. Thus, the relationship between sleep duration and ADL was partially mediated by these two factors (**Figure 2**, **Figure 3**).

## DISCUSSION

Our results showed that depressive symptoms and cognitive function significantly mediate the effect of sleep duration on ADL. Specifically, all three mediating pathways were significant: depressive symptoms alone mediated the effect, cognitive function alone mediated the effect, and depressive symptoms and cognitive function co-mediated the effect. These findings highlight the importance of focussing on both depressive symptoms and cognitive function in older adults.

A more important finding is the complex mechanism of sleep duration in older adults, as there is little definitive research on the relationship between sleep duration and cognitive function. Previous studies have also shown that nighttime sleep that is too long, too short, or both can lead to cognitive decline [49-52]. We found no significant linear correlation between sleep duration and cognitive function. However, the impact of sleep duration on cognitive function was negative when sleep duration was >8 hours. This finding provides a comprehensive warning regarding the prevention of cognitive decline, emphasising that rational sleep schedule regulation may be a key strategy for protecting against it.

We found that depressive symptoms mediated the relationship between sleep duration and ADL, indicating that increased sleep duration can enhance daily living abilities by reducing depressive symptoms. The results here are consistent with previous findings that sleep durations of 6–8 and ≥8 hours are protective factors regarding the occurrence of depressive symptoms in older adults compared to sleep durations of ≤6 hours [53,54]. Sleep can protect the brain, improve mood, and stabilise mental status. People with shorter sleep duration often experience insomnia, and insomnia patients frequently have poor mental health [55]. Consequently, this will lead to the occurrence of insomnia and sleep deprivation, forming a vicious circle, indicating the importance of maintaining adequate sleep. Studies have shown that depressive symptoms play a strong mediating role in the association between sleep duration and ADL. This is because sleep in older adults reduces depressive symptoms, and a better mental state helps maintain a stronger ability to perform ADL, which is consistent with previous findings that depressive symptoms lead to a decrease in the ability to perform daily living [56,57].

In this serial multiple mediation analysis, cognitive function mediated the relationship between sleep duration and ADL across classifications, indicating that sleep duration affects cognitive function and, thus, daily living ability in older adults. In contrast to traditional sleep protection hypotheses, our results suggest potential harms of prolonged sleep: each additional hour of sleep improves cognition in the 6–8 hours range but turns negative in >8 hours. In the Chinese context, napping is common among older adults; however, those who sleep ≥8 hours at night still show risk, suggesting a unique hazard associated with excessive continuous sleep at night. Established cohort studies have shown that an increase in sleep duration occurs 6–10 years before a dementia diagnosis [58], which supports and complements the conclusion here that excessive sleep duration is detrimental to cognitive function and that early cognitive dysfunction often manifests as excessive sleep duration. This suggests that older adults should maintain adequate sleep within the recommended range and avoid excessive sleep.

In addition, we found that depressive symptoms and cognitive function jointly mediated sleep’s effects on ADL, indicating that depressive symptoms and cognitive functioning mediate the relationship between sleep duration and ADL. This suggests that depressive symptoms affect cognitive functioning and older adults’ abilities to perform ADL. Cross-sectional surveys and prospective cohort studies in ex-personal samples have shown that depression is negatively correlated with scores in the cognitive domains of orientation, memory, attention, and computation, and that older adults with depression show a faster rate of cognitive decline [59]. Cognitive decline is also an early manifestation of dementia. One of dementia’s typical characteristics is the gradual decline in the ability to perform ADL or even the complete loss of the capacity to live independently. The minor role of cognitive functioning compared to the mediating effect of depressive symptoms is related to the fact that telephone cognitive testing is insensitive to early decline and that cognition may not be dominant until later in the ADL decline [60]. Although cognitive function did not play a dominant role in this study, its statistical significance remains clinically valuable as an essential window for early intervention in the decline of daily living skills.

In summary, we verified all three major hypotheses as significant, providing a strong scientific basis for older adult healthcare management. First, the results emphasise the importance of sleep duration for older adult health. With too little or too much sleep, they have a greater likelihood and more risk factors for depressive symptoms and cognitive decline or even the development of Alzheimer disease. This results in the loss of the ability to perform ADL. Although sleep quality is an important dimension, our focus on sleep duration aligns with its established role in geriatric health policies. Future studies that combine duration and quality metrics may further explain these mechanisms. Second, the results highlight the need for interventions that address depressive symptoms. Depressive symptoms not only affect older adults’ mental health but may also exacerbate cognitive decline and lead to the loss of the ability to perform ADL. Regular mental-health assessments are recommended to provide appropriate psychological and social support. This can be achieved by establishing community support networks, providing psychological counselling services, and mental health education activities for older adults. Third, our results showed that protecting cognitive function cannot be ignored, as it is another important mediator of sleep duration and ADL. Older adults, especially those with excessive sleep duration, should be encouraged to engage in activities that enhance cognitive function, such as social interaction and exercise. Appropriate interventions should prevent cognitive decline, which can be organised and facilitated through community centres, older adult activity groups, or online platforms. Finally, the effectiveness of integrated intervention strategies is relevant. The total effect of our mediation model was greater than 60% for both older adults with 6–8 or ≥8 hours of sleep. This effect has clear public health value. The government should develop and implement multidimensional intervention programmes and formulate more accurate and scientific ones based on comprehensive management of sleep education, mental health promotion, and cognitive function training. Placing the ‘main focus on depression and cognition’ approach should also be explored. Based on the grading of effect sizes in this study, we suggest prioritising the implementation of depression screening and intervention, as well as cognitive monitoring for older adults who sleep >8 hours.

This study had some limitations. First, we did not assess sleep quality owing to data constraints. Future research should integrate both duration and quality measures to capture the multidimensional effects of sleep. The second limitation is the cross-sectional design. Cross-sectional studies limit inferences of causality, and prospective studies should be conducted to achieve longitudinal changes in test outcome measures. Third, some self-reporting limitations existed. We will delete extreme sleep report values (<4 or >10 hours), multiple interpolation of sleep duration, and use sleep, depression, and cognition as latent variables to correct measurement errors using structural equation modelling.

## CONCLUSIONS

We provide important insights into the scientific and social implications of the relationship between sleep duration and ADL. Our results emphasise the importance of rationally regulating sleep duration, managing depressive symptoms, and enhancing cognitive function in maintaining older adults’ ability to live their daily lives. We also provide new perspectives and strategies for managing health careers. By focussing on sleep duration, depressive symptoms, cognitive function protection, and comprehensive intervention strategies for older adults, we can better address the challenges posed by population ageing, improve their quality of life, and provide them with more effective support and security. In the context of China's ‘silver wave’, sleep screening should be included in the annual physical examination of people over 65 years, and combined sleep-depression-cognition screening should be included in elderly health management. Personalised programmes can be implemented precisely for different sleep subgroups. Among those sleeping ≥8 hours, the negative impact on cognitive function suggests a potential risk of neurodegeneration, warranting increased vigilance. In this group, sleep regulation should be paired with cognitive training. In contrast, for individuals sleeping ≤6 hours, depressive symptoms emerge as the primary concern, and interventions should prioritise mental health support. Additionally, village doctors and primary caregivers can be trained to use simplified versions of the Center of Epidemiological Studies Depression Scale, ten-item version, and Telephone Interview for Cognitive Status scales to scientifically detect depressive symptoms and cognitive functioning. Cognitive training can also be provided to those with ≥8 hours of sleep and ≤15 cognitive status telephone interview scores, to practically promote older adult health.

## Additional material


Online Supplementary Document
