# Supplementary material for: The impact of sleep duration, depressive symptoms, and cognitive function on daily activity among Chinese older adults: a serial multiple mediation model
Source: J Glob Health. 2025 Sep 12;15:04267. doi: 10.7189/jogh.15.04267 (PMC12427602; doi:10.7189/jogh.15.04267)
Supplement: Online Supplementary Document [file jogh-15-04267-s001.pdf]

Supplement to: Sun J, Li H, Liu B, Deng J, Liu J, Hu N, Liu H, Shi L. The impact of sleep duration, depressive symptoms, and cognitive function on daily activity among Chinese older adults: a serial multiple mediation model. J Glob Health. 2025;15:04267.

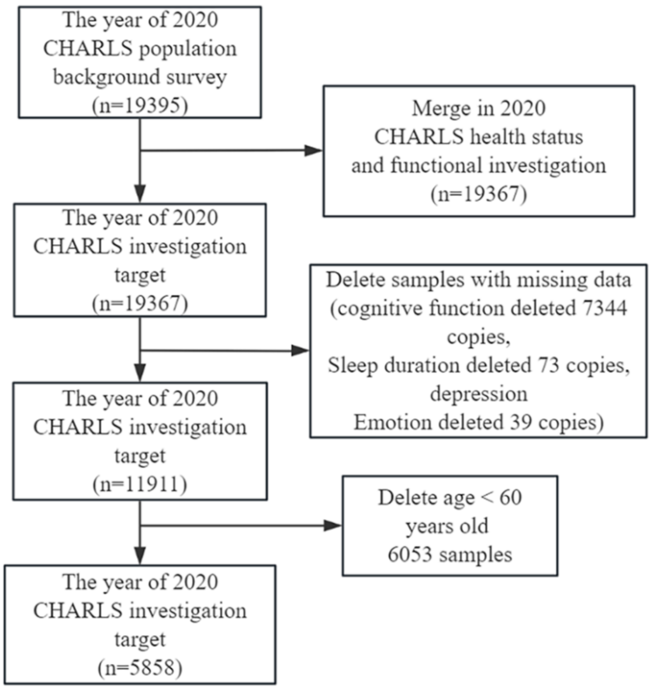

Figure S1. Flowchart of study sample inclusion

Table S1. Correlation analysis of different study variables

	ADL	Cognition	Depression	Sleep duration
ADL	1	0.157**	-0.338**	0.153**
Cognition	0.157**	1	-0.225**	0.019
Depression	-0.338**	-0.225**	1	-0.273**
Sleep duration	0.153**	0.019	-0.273**	1

**$p<0.01$
